# Molecular Cloning, Expression Profiling, and Marker Validation of the Chicken* Myoz3 *Gene

**DOI:** 10.1155/2017/5930918

**Published:** 2017-05-11

**Authors:** Maosen Ye, Fei Ye, Liutao He, Yiping Liu, Xiaoling Zhao, Huadong Yin, Diyan Li, Hengyong Xu, Qing Zhu, Yan Wang

**Affiliations:** Farm Animal Genetic Resources Exploration and Innovation Key Laboratory of Sichuan Province, Sichuan Agricultural University, Chengdu Campus, Chengdu, China

## Abstract

*Myozenin3* (*Myoz3*) has been reported to bind multiple Z-disc proteins and hence play a key role in signal transduction and muscle fiber type differentiation. The purpose of current study is to better understand the basic characteristics of* Myoz3*. Firstly, we cloned the ORF (open reading frame) of the* Myoz3* gene. AA (amino acid) sequence analysis revealed that the* Myoz3* gene encodes a 26 kDa protein which have 97% identities with that of turkey. Expression profiling showed that* Myoz3* mRNA is mainly expressed in leg muscle and breast muscle. Furthermore, we investigated* Myoz3* gene polymorphisms in two broiler breeds, the Yellow Bantam (YB) and the Avian. Five SNPs (single nucleotide polymorphisms) were identified in the YB breed and 3 were identified in the Avian breed. Genotypes and haplotype were constructed and their associations with carcass traits were analyzed. In the YB breed, c.516 C>T had a strong effect on both shank bone length and the $L^{⁎}$ value of breast muscle, and the H1H3 diplotype had the highest FC compared to other diplotypes. The markers identified in this study may serve as useful targets for the marker-assisted selection (MAS) of growth and meat quality traits in chickens.

## 1. Introduction

With the continuing growth of the economy and the rapid progress in the poultry industry in China, efforts to increase the growth rate of broilers are no longer the priority. Rather, consumers demand greater meat quality. However, meat quality is a complex trait influenced by genetic, nutritional [1], and environmental [2–4] factors. Muscle fiber type is an important factor influencing meat qualities [5]. Different proportions of slow-twitch (Type I) and fast-twitch (Types IIA, IIB, and IIX) muscle result in different meat characteristics, including colors/stability, tenderness, water holding capacities [6], and metabolic rates in the chickens [7].* Myozenin3* (*Myoz3)* is one of the candidate genes that play a key role in muscle fiber type specialization.

*Myoz3*, also known as* Calsarcin-3 *or* FATZ3*, belongs to a family of three members, consisting of* Myoz1 (Calsarcin-2), Myoz2 (Calsarcin-1)*, and* Myoz3*. In mice, Myozenin family members are predominantly expressed in skeletal and heart muscles [8], and* Myoz2 *is expressed in the heart muscle and specifically in the slow-twitch skeletal muscle [9, 10];* Myoz1* and* Myoz3 *are expressed only in skeletal muscle and are enriched in the fast-twitch muscle [11, 12]. Functional studies revealed that all three members of the Myoz family are able to influence the formation and maintenance of the Z-disc and interact with multiple Z-disc proteins including *α*-actinin, *β*-filamin, Telethonin, and Calcineurin [13]. Calcineurin is a calcium/calmodulin-dependent serine/threonine phosphatase [14], which is an important signaling molecule that is expressed in skeletal muscles, and it regulates the muscle fiber type by interacting with NFAT (nuclear factor of activated T cells) [15, 16].* Myoz1* KO mice displayed markedly improved performance due to enhanced Calcineurin signaling [17].* Myoz2, *similar to* Myoz1, *can regulate cardiac hypertrophy [18]. Mice with a null mutation in* Myoz2 *displayed an excess of slow skeletal muscle fibers due to the negative modulation of* Calcineurin *function [19].* Myoz2 *can also function as a protector against angiotensin-II-induced cardiac hypertrophy [20].

With the capacity to bind multiple proteins,* Myoz3 *has an important role in the regulation of Z-disc structure and signal transduction [21], resulting in muscle fiber differentiation. However, chicken* Myoz3 *has yet to be fully studied. The objectives of the current study were to better understand the characteristics of the* Myoz3* protein by cloning the ORF (open reading frame) of the* Myoz3* gene, to analyze the expression pattern of* Myoz3* mRNA in multiple tissues, and to investigate the correlation between* Myoz3 *gene polymorphisms and chicken carcass traits with the hope of providing markers for the MAS (marker-assisted selection) of economic traits and laying a foundation for future studies.

## 2. Materials and Methods

### 2.1. Ethics Statement

All the animal experiments were approved by the Committee on Experimental Animal Management of Sichuan Agricultural University and carried out strictly according to the Regulations for the Administration of Affairs Concerning Experimental Animals of the State Council of the People's Republic of China. Chickens involved in this study were executed as painlessly as possible to reduce their suffering.

### 2.2. Animals and Samples Collection

Avian and Yellow Bantam (YB) broiler breeds were used in the current study. 300 fertile eggs of the Avian were purchased from Wenjiang Zheng Da Corporation (Chengdu, Sichuan), and 300 fertile eggs of the Yellow Bantam breed were purchased from Jinling Animal Husbandry (Nanning, Guangxi). On the day that eggs were hatched, we randomly selected 44 males and 44 females from each breed. All the chickens were housed under the same environmental and nutritional conditions at the experimental farm for poultry breeding at Sichuan Agricultural University (Ya'an Sichuan).

To investigate the expression pattern of* Myoz3,* 3 embryos from each breed were executed on embryonic day 15 (E15) and 6 chickens (3 males and 3 females) from each breed were executed on day 1 (D1), day 40 (D40), and day 70 (D70). The liver, leg, heart, and breast muscles were immediately dissected from each embryo or chicken after execution and stored in liquid nitrogen until RNA extraction.

### 2.3. Measurement of Carcass and Meat Quality Traits

On day 70, 70 (35 male and 35 female) chickens from each breed were humanely executed by qualified technicians in a clean slaughter house using the clean neck cut method. During slaughtering, 24 carcass traits include semievisceration weight (SEW), evisceration weight (EW), breast muscle weight (BMW), leg muscle weight (LMW), wing weight (WW), heart weight (HW), liver weight (LW), gizzard weight (GW), proventriculus weight (PW), abdominal fat weight (AFW), body weight (BW), femoral weight (FW), shank bone weight (SW), metatarsal weight (MW), femoral diameter (FD), shank bone diameter (SD), metatarsal diameter (MD), femoral length (FL), shank bone length (SL), metatarsal length (ML), femoral circumference (FC), shank bone circumference (SC), metatarsal circumference (MC). PH value, lightness (*L* ^*∗*^), redness (*a* ^*∗*^), and yellowness (*b* ^*∗*^) of breast and leg muscles were also measured. On the following analysis, all weight-related traits were first analyzed before normalization and then normalized by dividing individual body weight (BW), for example, BMW (breast muscle weight) ratio = BMW/BW. Venous blood from each chicken was collected prior to execution and store at −20°C before DNA extraction.

### 2.4. DNA and RNA Extraction

Genomic DNA was extracted from venous blood using the standard phenol/chloroform method. The concentration and purity of the genomic DNA were measured using Nanodrop (Thermo Scientific, USA). Tris-EDTA buffer was added to the DNA samples to produce a final concentration of 100 *η*g/*μ*L. The DNA samples were maintained at −20°C until use.

Total RNA was extracted from the liquid nitrogen-frozen tissues described above, using Trizol reagent (Invitrogen, USA). After being extracted, total RNA was treated with RNase-free H2O (Tiangen, China). The concentration of the extracted RNA was measured using the Nanodrop2000 (Thermo Scientific, USA). First-strand cDNA was synthesized from 1 *μ*g of total RNA using the PrimeScript1 RT Reagent Kit (Perfect Real-Time) (TaKaRa, Biotechnology Co. Ltd., Dalian, China). The reactions were performed under the following conditions: 42°C for 2 min, 37°C for 15 min, and 85°C for 5 sec. The reactions were then stored at 4°C as described in the manufacturer's instructions.

### 2.5. Cloning and Sequence Analysis of Chicken* Myoz3* Gene

Based on the* Myoz3* transcript sequence published in the Ensemble database (EMBL ID: ENSGALG00000004560), two pairs of primers were designed with Primer Premiers 5 to clone the ORF (open reading frame) of the* Myoz3* gene. RT-PCR (reverse transcript PCR) amplification was performed in 50 *μ*L reaction volumes using the following cycling conditions: an initial step of denaturation for 5 min at 95°C; 31 cycles of 35 s at 95°C, 35 s at 55°C or the annealing temperature of the primers used (Table 1), and 40 s at 72°C; and a final extension step for 10 min at 72°C. The PCR products were purified with a gel extraction kit (Takara, Dalian, China) and sequenced by Tsingke Biological Technology (Chengdu, Sichuan). Based on the sequencing results, we conduct series of bioinformatics analyses. First, we thought to analyze the phylogeny relationship of Myoz3 protein among 13 species. Neighbor-joining method tree was constructed using MEGA5 and DNASTAR software. Next, we predicted several basic characteristics using ProtParam (http://us.expasy.org/tools/protparam.html), including its molecular weight, theoretical pI, amino acid composition, atomic composition, extinction coefficient, estimated half-life, instability index, and aliphatic index. Then we predicted its hydrophobicity or hydrophilicity using ProtScale (http://www.expasy.org/cgi-bin/protscale.pl) website. To predict its transmembrane helices and coils, we use TMHMM website (http://www.cbs.dtu.dk/services/TMHMM) and COILS websites (http://www.ch.embnet.org/software/COILS_form.html) [22]. Subcellular location was predicted with TargetP (http://www.cbs.dtu.dk/services/TargetP/) [23]. After basic characteristics prediction, we thought to construct 3D structure of Myoz3 protein, first we use CPHmodels, a website based on its template recognition function on profile-profile alignment guided by secondary structure and exposure predictions, to predict protein structure, and then we apply Pymol software (version 1.5) [24] to further enhance the 3D structure predicted in former step.

### 2.6. Expression Pattern Analysis of Chicken* Myoz3* Gene

Expression pattern of* Myoz3* gene was detected by RT-qPCR (real-time quantitative PCR). Based on cloning results, two pairs of primers were designed with Primer Premier 5 (Table 2).* GAPDH* was chosen as the housekeeping gene for normalization. An 11 *μ*L reaction system containing 6 *μ*L of SYBR premix Ex TAq™ (Takara), 1 *μ*L of cDNA, 0.5 *μ*L of forward primer, 0.5 *μ*L of reverse primer, and 3 *μ*L of RNase-free H_2_O (Tiangen) was used for RT-qPCR. Reaction was carried out using the PCR touch T960 (Hangzhou Jingle Scientific instrument Co., Ltd.) with the following amplification conditions: 95°C for 10 s followed by 40 cycles of 95°C for 5 s and 60°C for 30 s. Each sample was run in 3 duplicates. The 2^−ΔΔCt^ method was applied to analyze* Myoz3* mRNA expression.

### 2.7. Detection of Chicken* Myoz3* SNPs and Genotyping

Four primer pairs were used to detect polymorphisms of the* Myoz3 *gene exon. The primers were designed with Primer Premiers 5 based on the chicken* Myoz3* sequence (EMBL ID: ENSGALG00000004560) and were synthesized by Tsingke Biological Technology (Chengdu, Sichuan) (Table 3). A total of 6 DNA pools (3 pools from each breed) made up of 30 or 10 DNA samples were constructed to detect SNPs in* Myoz3*. A 25 *μ*L reaction containing 2.5 *μ*L of pooled DNA, 1.25 *μ*L (10 pmol/*μ*L) of each primer, and 12.5 *μ*L of 2×Mastermix (consisting of Mg2+, dNTPs, Taq DNA polymerase, and 7.5 *μ*L of ddH_2_O) was used for PCR. The PCR amplification protocol was as follows: an initial step of denaturation for 5 min at 95°C; 31 cycles of 35 s at 9°C, 35 s at 55°C (or the annealing temperature of the primers used as shown in Table 3), and 40 s at 72°C; and a final extension step for 10 min at 72°C. The PCR products were purified with a gel extraction kit (Takara, Dalian, China) and sequenced by Tsingke Biological Technology (Chengdu, Sichuan). The sequences were analyzed with the DNASTAR and MEGA5 [25] software.

After analyzing the results, we found polymorphic sites on exon2, exon4, and exon5. Then, we amplified the genomic DNA extracted from each chicken using the PCR system with protocol described above. The products were sequenced by Tsingke Biological Technology (Chengdu, Sichuan). The genotyping was performed with the DNASTAR and MEGA5 software.

### 2.8. Statistics Analysis

The Hardy-Weinberg equilibrium was evaluated using the *χ* ^2^ test. Linkage disequilibrium (LD) was measured by *D*′ and *r* ^2^ and was determined using the Haploview4.1 software [26]. Allelic frequencies were determined by directly counting the observed genotypes. The general linear model (GLM) procedure of SAS 9.4 (Statistical Analysis Systems Institute Inc., Cary, NC) was used to test the associations between the genotyped markers and carcass traits. The model is as follows:

(1) $$\begin{array}{c} Y_{ijk}=\mu +S_{i}+G_{j}+B_{k}+G_{j}\times S_{i}\times B_{k}+E_{ijk}, \end{array}$$

where *Y*_*ijk*_ is the measured trait; *μ* is the population mean; *S*_*i*_ is the fixed effect of sex; *G*_*j*_ is the fixed effect of genotype; *B*_*k*_ is the fixed effect of breed; *G*_*j*_ × *S*_*i*_ × *B*_*k*_ is the interaction among genotype, sex, and breed; and *E* is the random error.

One-way ANOVA was used to examine differences in* Myoz3* mRNA expression. All the values were considered significant at *P* < 0.05 and are presented as the mean ± SE.

## 3. Results

### 3.1. Cloning and Sequence Analysis of* Myoz3*

**Figure 1:**
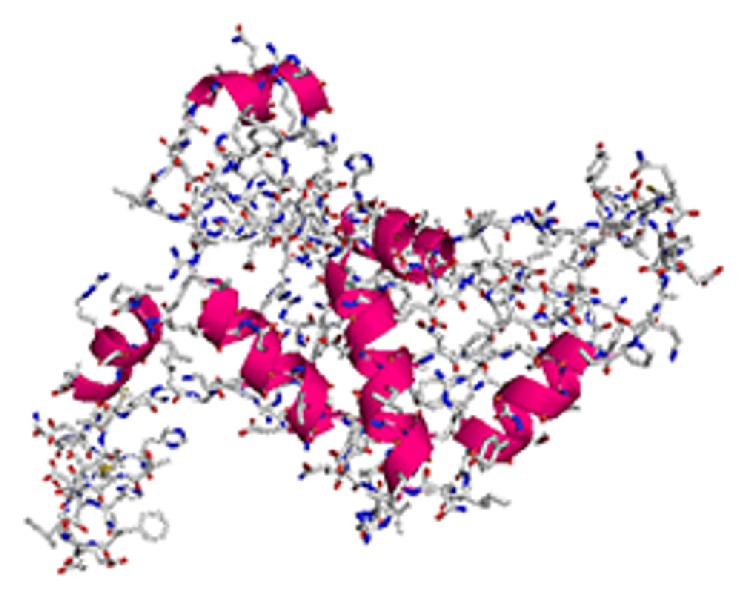
3D structure of the Myoz3 protein.

**Figure 2:**
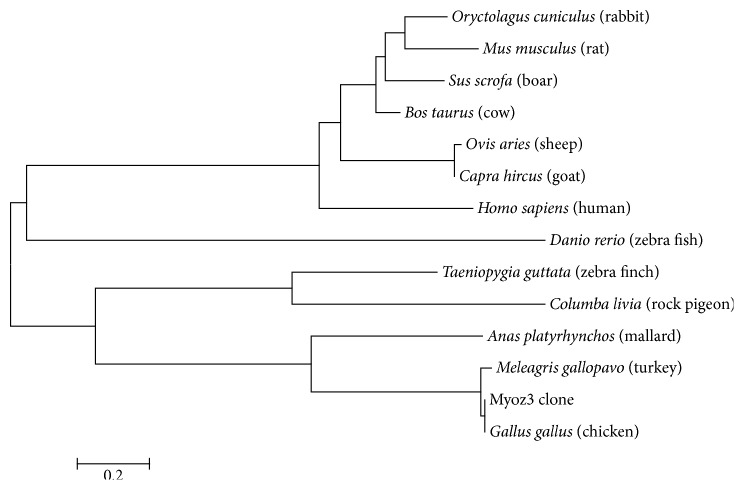
Neighbor-joining phylogeny of the Myoz3 protein.

By RT-PCR, we were able to clone the complete 726 bp ORF of the* Myoz3* gene.* Myoz3* gene encodes a protein containing 241AA and maps to a single domain named Calsarcin, which extends from AA1 to AA239 (see S1 Fig in Supplementary Material available online at https://doi.org/10.1155/2017/5930918). The theoretical mean molecular weight and the isoelectric point (pI) of the Myoz3 protein were 26755.2 Da and 6.51. The theoretical extinction coefficient was 22460. Next, we predicted the hydrophobicity and hydrophilicity scales (S2 Fig). The subcellular location prediction indicates all 241AA localized within the cell. No signal peptide or coil or transmembrane helices were predicted (S3–S5 Figs). The motif prediction revealed 14 motifs on the* Myoz3* protein including an endoplasmic reticulum-targeting sequence (S1 Table). After analyzing the basic characteristics of* Myoz3*, we constructed its 3D structure (Figure 1) and NJ phylogeny (Figure 2) based on its AA sequence.

### 3.2. Expression Profiling of* Myoz3*

**Figure 3:**
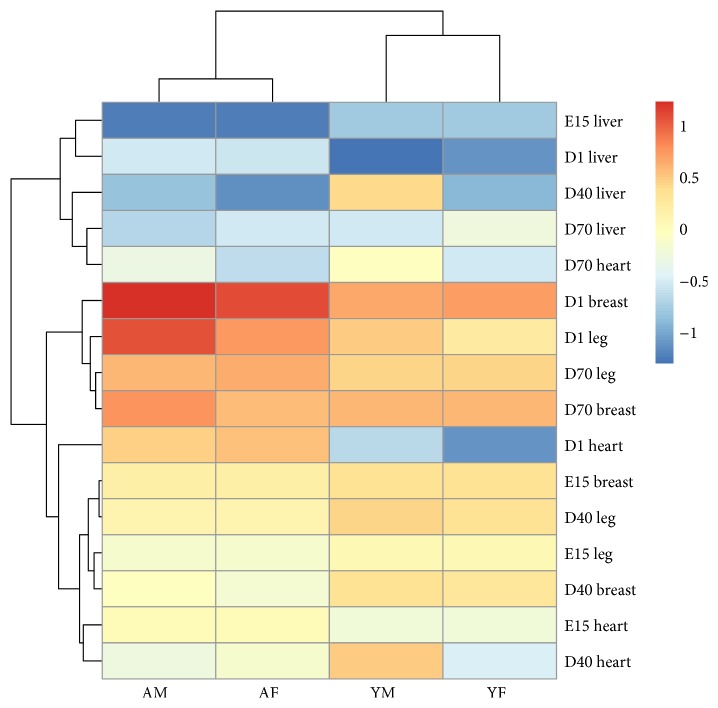
Overall presentation of profiled expression pattern of Myoz3. The data presented were log_10_ relative expression level of* Myoz3* mRNA as analyzed by RT-qPCR. AM stands for Avian male, AF stands for Avian female, YM stands for Yellow Bantam male, and YF stands for Yellow Bantam female.

**Figure 4:**
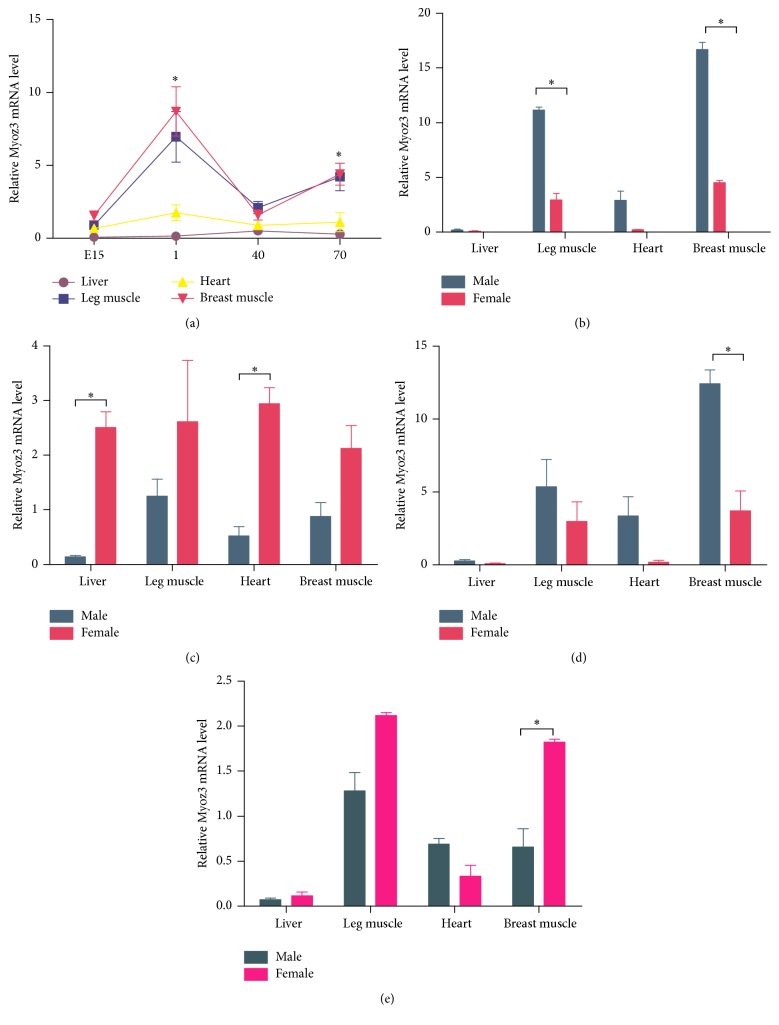
(a) Expression profiling in different tissues and at different times. E15 stands for embryonic day 15, 1 stands for 1 day after hatching, 40 stands for 40 days after hatching, and 70 stands for 70 days after hatching. (b) Expression profiling on embryonic day 15. (c) Expression profiling on 1 day after hatching. (d) Expression profiling on 40 days after hatching. (e) Expression profiling on 70 days after hatching. The error bars represent SEM; all experiments were replicated three times. ^*∗*^ *P* < 0.05.

The RT-qPCR data showed that* Myoz3 *was expressed from E15 to D70 in all the tissues tested, including the liver, leg muscle, heart muscle, and breast muscle (Figure 3). From E1 to D1, the expression level of* Myoz3* was significantly increased in the leg and breast muscle (*P* < 0.05) in both breeds. This was followed by a significant decrease from D1 to D40 (*P* < 0.05) and then stabilization from D40 to D70. No significant change of expression level was detected in the heart or liver. It is worth noting that* Myoz3* expression reached its peak on D1 in the liver, leg muscle, heart, and breast muscle (Figure 4 **)**. On D70, the expression level of* Myoz3* in the leg and breast muscle was significantly (*P* < 0.05) higher than that in the heart and liver.

Sex was found to be a major factor to influence the expression level of* Myoz3* in different tissues. In the Avian breed, expression level of* Myoz3 *in the leg and breast muscles was significantly higher (*P* < 0.05) among males on D1 (Figure 4). In contrast, the expression of* Myoz3* in the heart and liver was significantly higher in females on D40 (Figure 4). No significant (*P* > 0.05) difference in expression was found between male and female on E15 or D70. The Yellow Bantam breed showed the same pattern. On D1, the expression of* Myoz3* in male breast muscle was significantly (*P* < 0.05) higher than in female breast muscle (Figure 4). However, on D40, the same tissue showed the opposite result, with the expression level of* Myoz3 *being significantly (*P* < 0.05) higher in female breast muscle (Figure 4). The same pattern in the two breeds suggests that the expression pattern is not likely breed-specific.

### 3.3. Detection of SNPs in the* Myoz3* Gene

The results of the PCR amplification and direct sequencing were compared with the* Myoz3* gene sequence published in Ensemble (ID: ENSGALG00000004560). We found 5 variations (c.240C>T, c.501C>T, c.516C>T, c.576C>T, and c.675C>G) of the* Myoz3* gene in the Yellow Bantam breed and three (c.240C>T, c.576C>T, and c.675C>G) in the Avian breed. c.240C>T was located on exon 2; c.501C>T, c.516C>T, and c.576C>T were located on exon 4; and c.675C>G was located on exon 5. The CC genotype was the most common homozygous allele among all the SNPs. None of the SNPs of the two breed caused an amino acid change.

**Figure 5:**
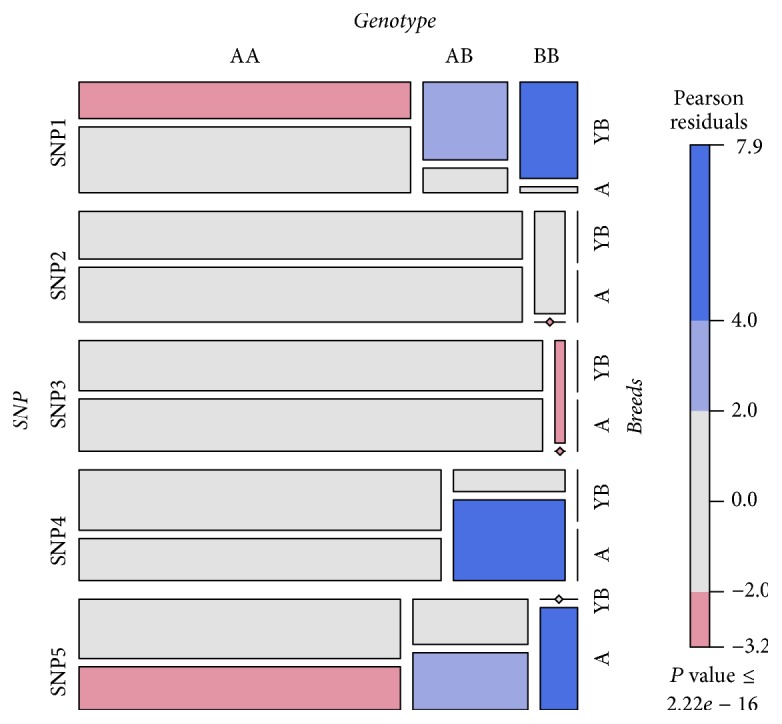
Distribution of genotype in two breeds. A stands for Avian breed, YB stands for Yellow Bantam breed, AA stands for major allele homozygote, AB stands for heterozygote, and BB stands for the minor allele homozygote.

The frequencies of the genotypes and alleles of the* Myoz3* gene in the two breeds were calculated (Table 4 and Figure 5). Because there was a vast difference between the two breeds, we calculated the frequencies of the genotypes and alleles separately.

### 3.4. Haplotype and Diplotype Construction and Frequencies

The haplotype and diplotype for the two breeds were constructed separately. SNP c.240C>T in the Yellow Bantam breed and SNP c.675C>G in the Avian breed did not conform to the Hardy-Weinberg equilibrium (*P* < 0.05) and were excluded. Five haplotypes were constructed in the Avian breed, and 4 were constructed in the Yellow Bantam breed. Based on the constructed haplotypes, 5 and 4 diplotypes were constructed in the Avian and Yellow Bantam breed, respectively (Table 5).

### 3.5. Analysis of the Association between Markers and Carcass Traits

An analysis of the association between the SNPs and carcass traits revealed that only two SNPs were associated with carcass traits in the YB breed (*P* < 0.05). SNP c.501C>T had a strong effect on FC (Table 6). Chickens with the CC genotype were significantly advanced compared to those with the CT genotype. With regard to SNP c.516C>T, chickens with the CC genotype had a longer SL and a lower *L* ^*∗*^ value of breast muscle compared to those with the CT genotype; both of these factors are considered to be favorable carcass traits. No significant correlation between the SNPs and carcass traits was found in the Avian breed.

After excluding haplotype frequencies lower than 0.02, an association analysis between haplotype/diplotype and traits revealed that, in the Yellow Bantam breed, the haplotype H3 is significantly associated with wing weight ratio (WWR); individuals marked by H3 haplotype had lower WWR compared to those who were not marked by H3. Chickens marked by haplotype H4 had lower shank bone weight ratio (SWR), and the H1H3 diplotype had a higher FC compared to other diplotypes (Table 6). In avian line, individual marked by haplotype H2 had higher ratio compare to those who were not. No significant correlation between diplotype and carcass traits was found in the Avian breed.

## 4. Discussion

Studies conducted on* Myoz3* have mainly been focused on its negative role in the regulation of* Calcineurin* [9, 12, 21, 27], a phosphatase that can control muscle fiber differentiation. The activation of* Calcineurin* in skeletal muscle selectively upregulates slow-fiber-specific gene promoters [13], allowing* Myoz3 *to play a key role in muscle fiber differentiation. Muscle fiber type is an important factor that influences meat quality [5] and can influence the rate of metabolism in the chicken. Although studies focused on chicken* Myoz3 *are of great importance and interest, they are currently lacking. In the present study, we dedicated our work to studying* Myoz3* by analyzing the sequence of the* Myoz3 *ORF and protein, the expression pattern of* Myoz3* mRNA in different tissues and* Myoz3* gene polymorphisms, and their association with carcass traits.

To better understand the basic characteristics of* Myoz3*, we cloned the* Myoz3 *ORF and analyzed the AA sequence of the Myoz3 protein. Multiple features were predicted including the theoretical mean molecular weight, pI, and extinction coefficient. The motif prediction revealed 14 motifs within the Myoz3 protein, including an endoplasmic reticulum-targeting sequence at the last 4 AA, which indicates that the protein may permanently reside in the lumen of the endoplasmic reticulum. It has been reported that the last 5 AA of Myoz3 serve as a motif for binding members of the PDZ domain protein family. This was based on the finding that the truncated protein was unable to bind the PDZ domain family members, while the WT protein was able to bind them [12]. In present study, we found that the last 5 AA are consistent with those found in humans (E [ST] [DE] [DE]L). This prediction is not the most accurate means to analyze the motifs or domains residing in the Myoz3 protein. Thus, further studies are needed to screen for proteins that interact with chicken Myoz3. A putative evolutionary distance analysis carried out by constructing a NJ phylogenetic tree suggests that the evolutionary distance of Myoz3 is similar to that of other species, indicating a relative conserve function among species.

The expression pattern of* Myoz3 *gives us insight into its function. Expression profiling in porcine revealed a trend where by the expression level of* Myoz3 *rose throughout the prenatal and postnatal development periods in skeletal muscle [28]. In goat, analysis of the spatial mRNA expression pattern revealed that* Myoz3* was found mainly in abdominal muscles, leg muscles, the lungs, and the kidneys. Low expression levels were found in the spleen, and very little expression was detected in the heart and liver [29]. The expression pattern of* Myoz3* is also related to mutations in other muscle-specific genes such as ACTN3; the upregulation of* Myoz3 *was identified in human carriers of the ACTN3 R577X polymorphism [30]. Differences in the expression pattern among the three members of the Myoz family may also be due to the activation of different promoters [31]. In the current study, we found that* Myoz3* mRNA was predominantly expressed in leg and breast muscles. Less (*P* < 0.05) was detected in the heart and liver, which is consistent with the pattern identified in goat and mice. It has been reported that chicken breast muscle is mainly made up of fast-twitch muscle fiber and that leg muscle contains a higher proportion of slow-twitch muscle fiber [32], even though our results did not show that expression level of* Myoz3* was statistically significant higher in breast muscle, a trend that* Myoz3 *level is higher in breast muscle than that in leg muscle was observed, which is consistent with what has been observed in mammals. Distinguishing muscle fiber type is a complex and time-consuming process. The muscle fiber type-specific expression pattern of* Myoz3* suggests that* Myoz3 *is another potential marker for the rough identification of the proportion of muscle fiber type. However, the accuracy and efficiency of this marker need to be further validated. The temporal mRNA expression patterns were similar in the different breeds. Although there are significant differences between the genetic backgrounds of the Avian and Yellow Bantam breeds, our results suggest a relatively conserved role for* Myoz3* in chicken muscle development in the breeds. The different expression patterns in males and females may be due to the differences in the pattern of muscle development between the sexes. Whether the functions of* Myoz3* are different between the sexes needs to be further investigated.

Polymorphisms in the* Myoz3 *gene have a great potential to qualify as markers for MAS (marker-assisted selection), even though in porcine, the silent mutation T595C had no significant association with carcass traits [29]. In the current study, we identified 3 SNPs in the* Myoz3* gene in the Avian breed and 5 SNPs in the Yellow Bantam breed by direct sequencing. All the SNPs were synonymous mutations, which was hardly a surprise considering that* Myoz3 *is capable of binding multiple Z-disc proteins and that mutations in several proteins that are either Z-disc components or factors that bind to Z-disc proteins are thought to impact the physiology and pathology of muscle fiber [21]. Any nonsynonymous mutation has a considerable potential to impact the function of the* Myoz3 *protein. There is evidence to suggest that synonymous mutations can affect splicing and/or mRNA stability [33] and that they are correlated with the level of gene expression [34]. Thus, the synonymous mutations found in the current study may have an impact on the function of* Myoz3 *and may influence the carcass traits measured. Consist with our hypothesis, among the 5 SNPs found in the Yellow Bantam breed, c.516C>T is correlated with the *L* ^*∗*^ value of breast muscle. Considering the roles of* Myoz3* in muscle, it is not surprising that individuals with different genotypes display significant differences in regard to the lightness of breast muscle. However, the specific role of mutation in the diversity of breast muscle color needs to be clarified. All 3 SNPs found in the Avian breed were also found in the YB breed. Interestingly, the two SNPs found to correlate with carcass traits in the Yellow Bantam breed were not found in the Avian breed. Because the Avian breed is a well-bred broiler breed under relatively high selective pressure, we cannot rule out the possibility that these two SNPs were not identified due to the phenotype-based selection. Haplotype information is an essential component of many analyses of fine-scale molecular-genetics data [35]. In addition, the diplotype can identify more precise and distinct signals compared with single-locus tests [36]. We constructed 4 haplotypes and 5 diplotypes in the Avian breed and 4 haplotypes and 4 diplotypes in the YB breed. In the YB breed, the H1H3 diplotype was correlated with FC. In general, the markers found in this study can be considered to be practical for breeding and selection.

In conclusion, we understand the basic features of the Myoz3 protein and know that the expression pattern of* Myoz3 *in chicken is similar to that in mice and humans. Through temporal expression profiling, we revealed an age-specific expression pattern and determined that sex is a major factor that influences the expression of* Myoz3*. The analysis of the association between markers and carcass traits revealed multiple validated markers on* Myoz3* gene. The CC genotype of c.516C>T and the H1H3 diplotype could be used as potential advantageous molecular markers of carcass traits in the Yellow Bantam breed. Further work will be necessary to use this SNP for marker-assisted selection in different breeds and large populations.

## Supplementary Material

S1 Fig. Domain mapping of Myoz3. Myoz3 protein was mapped to one domain, Calsarcin. S2 Fig. Predicted hydrophilicity scales. The x axis represent position in Myoz3 protein, the y axis represents the hydrophilicity score. The negative score represents degree of hydrophobicity and positive score represent degree of hydrophilicity. S3 Fig. Signal peptide prediction. The x axis represent position in Myoz3 protein, the y axis represents the Signal peptide score. S4 Fig. Coils prediction. The x axis represent position in Myoz3 protein, the y axis represents the coils prediction score. S5 Fig. Transmembrane helix prediction. The x axis represent position in Myoz3 protein, the Y axis represent possibility of protein location, blue line represents the possibility of protein localized inside cell, the red line indicates the possibility of the protein's transmembrane potential and the pink line represent the possibility of protein localized outside of cell. S1 Table. Motifs mapped to Myoz3 protein. Location number represent amino acid index in Myoz3 protein.

## Figures and Tables

**Table 1:** Primers information.

Forward (5′-3′)	Reverse (5′-3′)	Product length (bp)	Annealing temperature (°C)	Usage
CCACCCAGTCGTCCTCAT	AGACCGCTAGGTTGACTTTC	905	57.6	Clone
TCTCAGCCAGAAATAACCG	CTGTCTTCTTGGGCACCTC	581	55.4	

Primers used to clone the ORF of Myoz3 gene.

**Table 2:** Primers information.

Forward (5′-3′)	Reverse (5′-3′)	Product length (bp)	Annealing temperature (°C)	Usage
CCAGAACATCATCCCAGCGTC	ACGGCAGGTCAGGTCAACAA	136	60	*GAPDH*
CCGAGGTGCCCAAGAAGA	GGGAATGGCGGTGATGTT	131	60	*Myoz3*

**Table 3:** Primers information.

Forward (5′-3′)	Reverse (5′-3′)	Product length (bp)	Annealing temperature (°C)	Amplify target
TCACCCACGTCCACCTTTCTC	TGTCCACTCGTTGGCTGCTC	567	57.6	Exon 1
GTGCAGCGTGCTCCAACAT	GGCATCTGTGGGACAAGGAG	577	58	Exon 2 3
GCAGCAAGAGGAAAGCCAGTG	GGATGGGTGGATGGAAAGATG	359	61.2	Exon 4
TTCCAGACCCGCTTTATCCA	ACGAGACCGCTAGGTTGACTT	372	61	Exon 5

Primers used to detection SNPs of Myoz3 gene.

**Table 4:** Genotype information.

SNP	Breed	Genotype frequencies			Allele frequencies		*P* value
		AA	AB	BB	A	B	
c.240C>T	A	0.90	0.09	0.01	0.94	0.06	0.08702
	YB	0.50	0.27	0.23	0.64	0.36	0.00053
c.501C>T	YB	0.87	0.13	0.00	0.94	0.06	0.56542
c.516C>T	YB	0.96	0.04	0.00	0.98	0.02	0.85463
c.576C>T	A	0.63	0.37	0.00	0.81	0.19	0.05637
	YB	0.90	0.10	0.00	0.95	0.05	0.65969
C.675C>G	A	0.57	0.27	0.16	0.71	0.29	0.00393
	YB	0.79	0.21	0.00	0.89	0.11	0.31538

A stands for Avian breed; YB stands Yellow Bantam breed; AA stands for major allele homozygote; AB stands for heterozygote; BB stands for the minor allele homozygote; *P* value is the results of *χ* ^2^ test of Hardy-Weinberg equilibrium.

**Table 5:** Haplotype and diplotype information.

Breed	Haplotype	c.240C>T	c.576C>T	Frequency	Diplotype	Frequency	
Avian	H1	C	C	0.768	H1H1	0.557	
	H2	C	T	0.175	H1H2	0.343	
	H3	T	C	0.046	H1H3	0.071	
	H4	T	T	0.011	H1H4	0.014	
					H3H4	0.014	

Breed	Haplotype	c.501C>T	c.516C>T	c.576C>T	Frequency	Diplotype	Frequency

YB	H1	C	C	C	0.864	H1H1	0.729
	H2	T	C	C	0.064	H1H2	0.129
	H3	C	C	T	0.05	H1H3	0.100
	H4	C	T	C	0.022	H1H4	0.043

YB stands for Yellow Bantam breed.

**Table 6:** Markers-carcass traits correlation in Yellow Bantam breed.

Breed	Markers	Traits	Value				*P* value
YB	Haplotype		H3 (7)		Non H3 (63)		
		WWR	32.63 ± 0.578		37.77 ± 4.886		0.032^*∗*^
	Haplotype		H4 (3)		Non H4 (67)		0.027^*∗*^
		SWR	6.67 ± 0.246		10.15 ± 0.869		

YB	Diplotype		H1H1 (51)	H1H2 (9)	H1H3 (7)	H1H4 (3)	
		FC	4.549 ± 0.044	4.300 ± 0.106	4.714 ± 0.120	4.200 ± 0.183	0.021^*∗*^
YB	SNP c.501C>T		CC (61)		CT (9)		
		FC	4.551 ± 0.042		4.300 ± 0.109		0.034^*∗*^

YB	SNP c.516C>T		CC (67)		CT (3)		
		*L* ^*∗*^	54.665 ± 0.421		58.939 ± 1.99		0.039^*∗*^
		SL	10.945 ± 0.184		9.118 ± 0.868		0.043^*∗*^

Avian	Haplotype		H2 (19)		Non H2 (51)		
		GWR	14.633 ± 0.266		13.288 ± 0.564		0.018^*∗*^

Markers that were associated with carcass traits; carcass traits are presented as least squares mean ± SEM. YB stands for Yellow Bantam. WWR stands for wing weight ratio; SWR stands for shank bone weight ratio; FC stands for femoral circumference; *L* ^*∗*^ stands for lightness of breast muscle; SL stands for shank length; GWR stands for gizzard weight ratio.
