# Supplementary figures and images for: Molecular Cloning, Expression Profiling, and Marker Validation of the Chicken Myoz3 Gene
Source: Biomed Res Int. 2017 May 11;2017:5930918. doi: 10.1155/2017/5930918 (PMC5444202; doi:10.1155/2017/5930918)

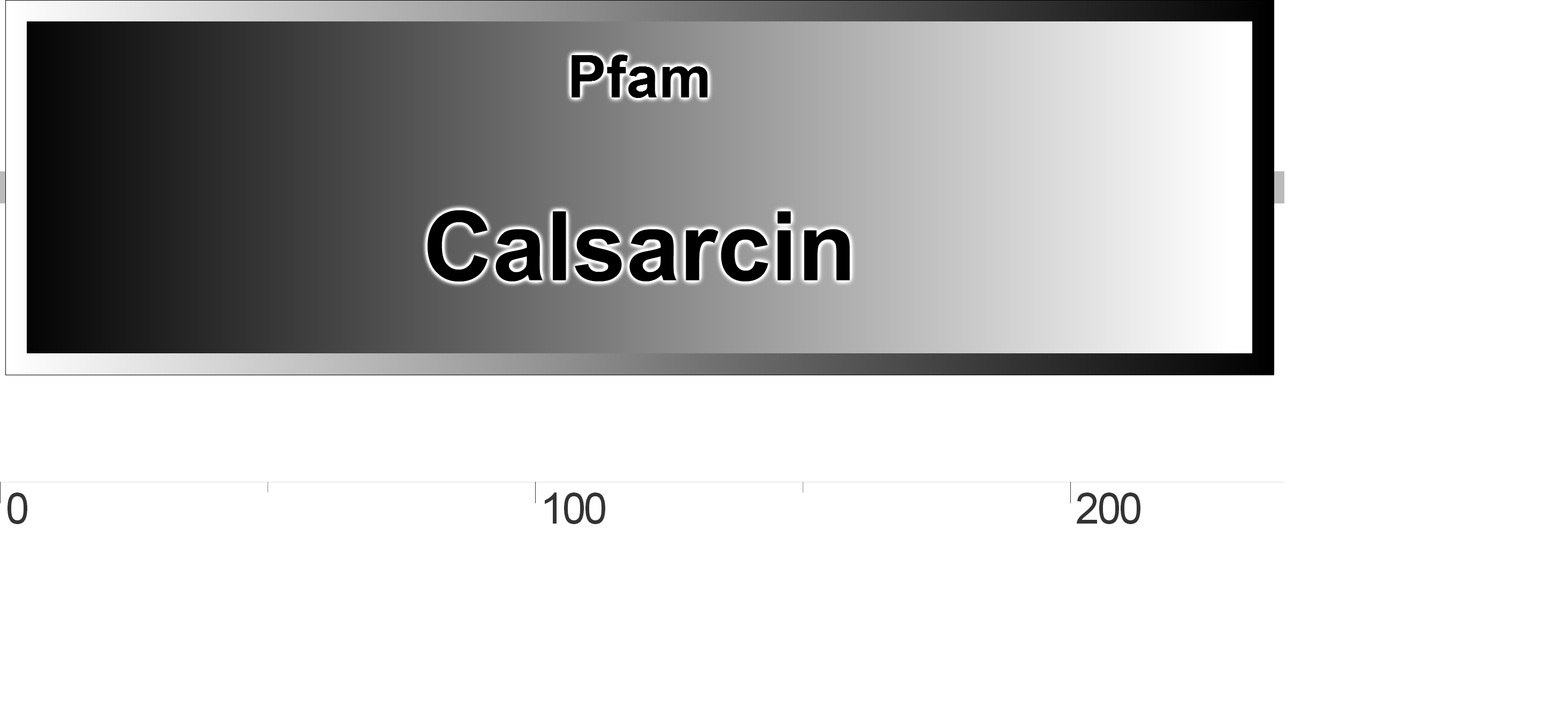

Supplement: Supplementary file 1 — S1 Fig. Domain mapping of Myoz3. Myoz3 protein was mapped to one domain, Calsarcin. S2 Fig. Predicted hydrophilicity scales. The x axis represent position in Myoz3 protein, the y axis represents the hydrophilicity score. The negative score represents degree of hydrophobicity and positive score represent degree of hydrophilicity. S3 Fig. Signal peptide prediction. The x axis represent position in Myoz3 protein, the y axis represents the Signal peptide score. S4 Fig. Coils prediction. The x axis represent position in Myoz3 protein, the y axis represents the coils prediction score. S5 Fig. Transmembrane helix prediction. The x axis represent position in Myoz3 protein, the Y axis represent possibility of protein location, blue line represents the possibility of protein localized inside cell, the red line indicates the possibility of the protein's transmembrane potential and the pink line represent the possibility of protein localized outside of cell. S1 Table. Motifs mapped to Myoz3 protein. Location number represent amino acid index in Myoz3 protein. [file 5930918.f1.zip › Supporting Information/S1_Fig.tif]

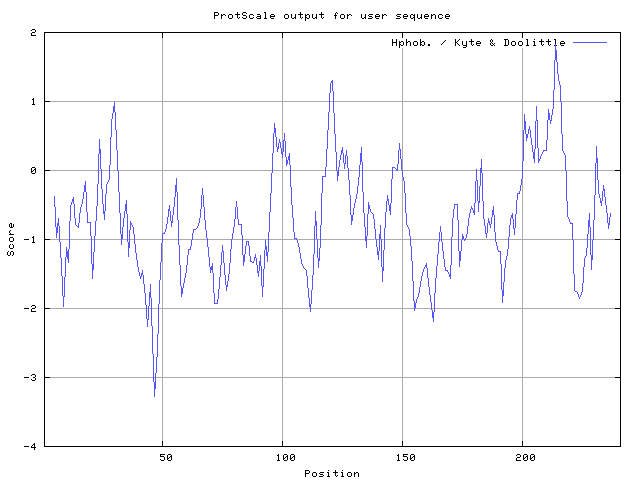

Supplement: Supplementary file 1 — S1 Fig. Domain mapping of Myoz3. Myoz3 protein was mapped to one domain, Calsarcin. S2 Fig. Predicted hydrophilicity scales. The x axis represent position in Myoz3 protein, the y axis represents the hydrophilicity score. The negative score represents degree of hydrophobicity and positive score represent degree of hydrophilicity. S3 Fig. Signal peptide prediction. The x axis represent position in Myoz3 protein, the y axis represents the Signal peptide score. S4 Fig. Coils prediction. The x axis represent position in Myoz3 protein, the y axis represents the coils prediction score. S5 Fig. Transmembrane helix prediction. The x axis represent position in Myoz3 protein, the Y axis represent possibility of protein location, blue line represents the possibility of protein localized inside cell, the red line indicates the possibility of the protein's transmembrane potential and the pink line represent the possibility of protein localized outside of cell. S1 Table. Motifs mapped to Myoz3 protein. Location number represent amino acid index in Myoz3 protein. [file 5930918.f1.zip › Supporting Information/S2_Fig.gif]

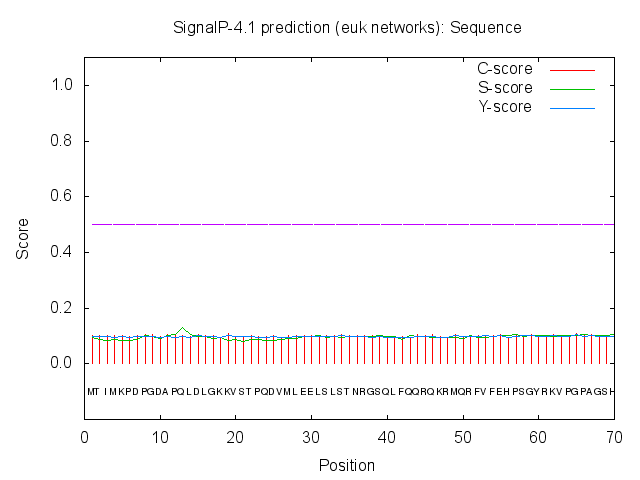

Supplement: Supplementary file 1 — S1 Fig. Domain mapping of Myoz3. Myoz3 protein was mapped to one domain, Calsarcin. S2 Fig. Predicted hydrophilicity scales. The x axis represent position in Myoz3 protein, the y axis represents the hydrophilicity score. The negative score represents degree of hydrophobicity and positive score represent degree of hydrophilicity. S3 Fig. Signal peptide prediction. The x axis represent position in Myoz3 protein, the y axis represents the Signal peptide score. S4 Fig. Coils prediction. The x axis represent position in Myoz3 protein, the y axis represents the coils prediction score. S5 Fig. Transmembrane helix prediction. The x axis represent position in Myoz3 protein, the Y axis represent possibility of protein location, blue line represents the possibility of protein localized inside cell, the red line indicates the possibility of the protein's transmembrane potential and the pink line represent the possibility of protein localized outside of cell. S1 Table. Motifs mapped to Myoz3 protein. Location number represent amino acid index in Myoz3 protein. [file 5930918.f1.zip › Supporting Information/S3_Fig.png]

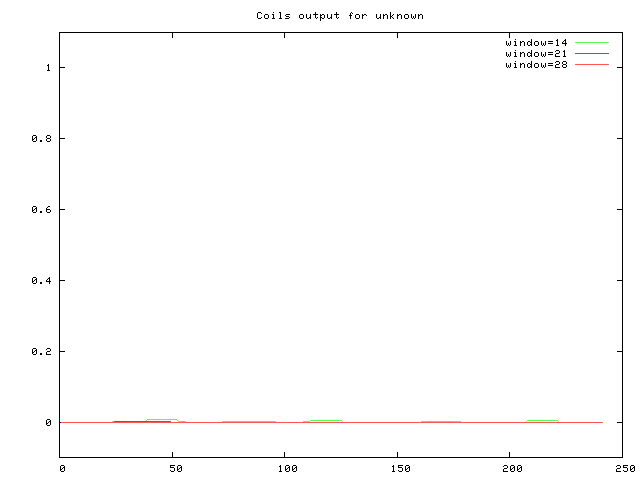

Supplement: Supplementary file 1 — S1 Fig. Domain mapping of Myoz3. Myoz3 protein was mapped to one domain, Calsarcin. S2 Fig. Predicted hydrophilicity scales. The x axis represent position in Myoz3 protein, the y axis represents the hydrophilicity score. The negative score represents degree of hydrophobicity and positive score represent degree of hydrophilicity. S3 Fig. Signal peptide prediction. The x axis represent position in Myoz3 protein, the y axis represents the Signal peptide score. S4 Fig. Coils prediction. The x axis represent position in Myoz3 protein, the y axis represents the coils prediction score. S5 Fig. Transmembrane helix prediction. The x axis represent position in Myoz3 protein, the Y axis represent possibility of protein location, blue line represents the possibility of protein localized inside cell, the red line indicates the possibility of the protein's transmembrane potential and the pink line represent the possibility of protein localized outside of cell. S1 Table. Motifs mapped to Myoz3 protein. Location number represent amino acid index in Myoz3 protein. [file 5930918.f1.zip › Supporting Information/S4_Fig.gif]

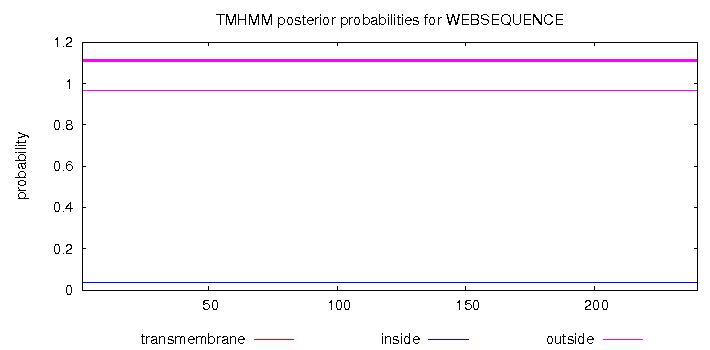

Supplement: Supplementary file 1 — S1 Fig. Domain mapping of Myoz3. Myoz3 protein was mapped to one domain, Calsarcin. S2 Fig. Predicted hydrophilicity scales. The x axis represent position in Myoz3 protein, the y axis represents the hydrophilicity score. The negative score represents degree of hydrophobicity and positive score represent degree of hydrophilicity. S3 Fig. Signal peptide prediction. The x axis represent position in Myoz3 protein, the y axis represents the Signal peptide score. S4 Fig. Coils prediction. The x axis represent position in Myoz3 protein, the y axis represents the coils prediction score. S5 Fig. Transmembrane helix prediction. The x axis represent position in Myoz3 protein, the Y axis represent possibility of protein location, blue line represents the possibility of protein localized inside cell, the red line indicates the possibility of the protein's transmembrane potential and the pink line represent the possibility of protein localized outside of cell. S1 Table. Motifs mapped to Myoz3 protein. Location number represent amino acid index in Myoz3 protein. [file 5930918.f1.zip › Supporting Information/S5_Fig.gif]
